# Swellable Microneedles in Drug Delivery and Diagnostics

**DOI:** 10.3390/ph17060791

**Published:** 2024-06-16

**Authors:** Hossein Omidian, Sumana Dey Chowdhury

**Affiliations:** Barry and Judy Silverman College of Pharmacy, Nova Southeastern University, Fort Lauderdale, FL 33328, USA; sd2236@mynsu.nova.edu

**Keywords:** swellable microneedles, drug delivery, diagnostics, biomedical monitoring, biocompatibility, sensor integration

## Abstract

This manuscript explores the transformative potential of swellable microneedles (MNs) in drug delivery and diagnostics, addressing critical needs in medical treatment and monitoring. Innovations in hydrogel-integrated MN arrays facilitate controlled drug release, thereby expanding treatment options for chronic diseases and conditions that require precise dosage control. The review covers challenges, such as scalability, patient compliance, and manufacturing processes, as well as achievements in advanced manufacturing, biocompatibility, and versatile applications. Nonetheless, limitations in physiological responsiveness and long-term stability remain, necessitating further research in material innovation and integration with digital technologies. Future directions focus on expanding biomedical applications, material advancements, and regulatory considerations for widespread clinical adoption.

## 1. Introduction

### 1.1. Overview of Polymers in Swellable Microneedles

Swellable gels possess a remarkable ability to swell significantly in aqueous environments, making them invaluable for various applications, particularly in the pharmaceutical field. The swelling capacity and mechanical stability of these gels can be meticulously engineered by manipulating factors, such as the chemical composition and crosslinking density of the polymers [1,2]. The versatility of swellable hydrogels extends to their ability to respond to environmental changes, such as temperature and pH, while generally being biocompatible. This further expands their utility in pharmaceutical and biomedical applications as swellable matrices, particles, and devices [3,4,5].

Swellable microneedles (MNs) represent a promising technology for advanced transdermal drug delivery, offering desired mechanical and biocompatible properties. The selection of polymers is crucial for ensuring the efficacy and safety of these microneedles. Specifically, hydrogel-based MN systems provide scalability in manufacturing, good insertion depth, and quality comparable to traditional methods [6]. Hydrogel-forming microneedle arrays (MNAs) are particularly effective for self-application, ensuring good skin penetration and reproducibility when applied by volunteers. Additionally, these MNAs can incorporate pressure-indicating sensors for visual feedback, enhancing their practicality and effectiveness [7,8].

2-Hydroxyethyl methacrylate (HEMA) and ethylene glycol dimethacrylate (EGDMA) are valued for their robust mechanical properties, light responsiveness, and prolonged drug delivery capabilities [9]. Similarly, formulations utilizing epigallocatechin gallate (EGCG) as a crosslinker with methotrexate (MTX) ensure H_2_O_2_-responsive behavior, dual-mode drug release, and prolonged skin retention [10]. Silk fibroin (SF), combined with phenylboronic acid and acrylamide, ensures biocompatibility, controllable degradability, glucose responsiveness, and sustained drug delivery [11]. Boronate-containing hydrogels with phenylboronic acid (PBA) exhibit temperature-independent glucose responsiveness, biocompatibility, and rapid, sustained release [12]. Chitosan is another cost-effective polymer known for its notable swelling capabilities, good degradability, and non-cytotoxicity [13].

Degradable crosslinked gels are characterized by their self-regulated, rapid responsiveness and excellent biocompatibility [14]. Polyvinyl alcohol (PVA) and polyvinylpyrrolidone (PVP), especially when combined with citric acid, demonstrate good swelling capacity, strong insertion capabilities, and non-toxicity. When mixed with hydroxypropyl methylcellulose (HPMC) and glycerol, these polymers offer sustained drug delivery, good mechanical strength, moderate swelling, and minimal side effects [15,16]. Agarose gel is suitable for 3D microfabrication and cell community studies due to its topographical control [17], while PVA is noted for its adjustable disassembly time, high permeability, and non-toxicity [18]. Dopamine (DA)–hyaluronic acid (HA) hydrogel, in conjunction with PEDOT:PSS and Ag-Pt nanoparticles, enables real-time, enzyme-less electrochemical sensing with enhanced electrical properties [19]. Photonic crystal hydrogels combined with phenylboronic acid facilitate non-invasive glucose detection, visual glucose responsiveness, and mobile phone integration [20].

### 1.2. Applications and Advancements of Swellable Microneedles

Nanometallic conductive composite hydrogels offer minimally invasive, real-time monitoring with a fast and linear response to glucose [21]. Meanwhile, poly(methyl-vinyl ether-co-maleic anhydride) and poly(ethylene glycol) enable minimally invasive extraction and rapid analyte uptake, making them ideal for diagnostic and monitoring purposes [22]. Methacrylated hyaluronic acid (MeHA) supports rapid interstitial fluid (ISF) extraction, structural integrity, and metabolic analysis [23]. Polyvinyl alcohol (PVA) combined with sodium alginate (SA) exhibits high oxidase-like activity, rapid extraction, and colorimetric detection [24]. The swellable bilateral core–shell structure allows for rapid absorption, selective reaction, and accurate lactate sensing [25]. Dopamine-conjugated hyaluronic acid (HA) and poly(3,4-ethylenedioxythiophene):polystyrene sulfonate (PEDOT:PSS) enable real-time pH measurement, increased conductivity, and high accuracy [26].

The versatility and effectiveness of these polymers in swellable microneedles underscore their potential for broad medical applications, ranging from drug delivery to real-time monitoring and diagnostics. These polymers’ unique properties, such as biocompatibility, mechanical strength, responsiveness to various stimuli, and ease of manufacturing, make them highly suitable for developing advanced transdermal drug delivery systems.

A significant advancement facilitated by microneedle technology lies in the field of advanced drug delivery systems. The incorporation of hydrogel technologies into MN arrays has enabled controlled release mechanisms that adapt to physiological changes, allowing for the on-demand delivery of therapeutic agents [9,10,11,12]. This has broadened the spectrum of treatable conditions, making it possible to administer drugs efficiently for chronic diseases, such as diabetes, and conditions requiring precise dosage control, such as psoriasis [10,11,13,14]. Hydrogel microneedles, celebrated for their exceptional biocompatibility and mechanical properties, are increasingly recognized as versatile tools in medical applications. They have been extensively evaluated for their design, preparation, and application in drug delivery, health monitoring, and wound healing. Their potential integration with smart devices presents a promising avenue for enhancing therapeutic management [5].

However, the transition from laboratory-scale production to commercial manufacturing of microneedle arrays presents significant challenges. Maintaining the integrity and effectiveness of these devices during large-scale production requires continuous research and innovation in production processes [6]. Additionally, the need for patient-friendly designs that can be safely and easily self-applied has driven advancements in MN technology aiming to ensure both safety and efficacy in home-use settings [7,8,27]. Enhancing patient compliance and comfort is crucial, with the development of non-invasive and pain-free application methods being particularly important. Swellable MNs offer a promising solution by reducing the invasiveness of traditional methods, thereby improving patient comfort and potentially increasing treatment adherence [11,15,16,28].

Innovations in the design and fabrication of microneedle arrays have focused on improving microfabrication processes to enhance the morphological stability and disassembly time of hydrogel-forming MNs. These improvements are essential for their practical application in cellular environments and for long-term use [17,18]. The demand for rapid and minimally invasive diagnostics has driven the development of microneedle technologies capable of continuous, real-time monitoring of biomarkers [29]. These devices are crucial for managing conditions, such as diabetes, where timely data can significantly influence disease management [19,20,21,22]. Moreover, the ability to swiftly detect biomarkers relevant to conditions like melanoma directly from interstitial fluid using microneedle arrays has the potential to change early diagnosis and treatment strategies [30]. Hydrogel microneedles are also being developed for applications requiring biofluid extraction, particularly in the food and agriculture sectors [31].

In summary, the use of swellable microneedle products addresses a wide array of gaps, needs, and challenges in the fields of drug delivery systems, patient compliance, commercial scalability, and advanced diagnostic capabilities. Each challenge continues to drive innovation in this evolving field, with the goal of providing more effective, user-friendly, and precise medical technologies

## 2. Drug Delivery Systems and Technologies

Microneedles are a transdermal drug delivery system that punctures the epithelium to deliver drugs directly to deep tissues, bypassing the first-pass effect of the gastrointestinal tract and reducing pain. Hydrogel microneedles, made from biocompatible hydrogels, offer controllable mechanical properties and drug release, and they can be modified for environmental control of drug release in vivo. The oral cavity’s large epithelial tissue is ideal for this method, as hydrogel microneedles can overcome mucosal barriers, ensuring drug efficacy despite the humid and dynamic environment [32].

Recent advancements in drug delivery systems include the development of crosslinked polymer microneedle arrays (MNAs) that create hydrogel conduits, forming direct pathways from drug reservoirs to the dermal microcirculation. This innovation enhances the transdermal delivery of macromolecules by enabling the microneedles to quickly absorb interstitial fluid while also allowing for easy sterilization. The adjustment of crosslink density in these microneedles improves drug delivery efficiency and effectiveness, offering a less invasive alternative for a variety of treatments [33]. Additionally, noteworthy progress in therapeutic applications is attributed to the development of hydrogel-forming microneedle arrays. These arrays are now manufactured more efficiently using a microwave-assisted crosslinking technique, which reduces production time without compromising drug delivery capabilities. This improved process has the potential to lower both costs and production times, benefiting the mass production of transdermal drug delivery systems [34].

**Figure 1:**
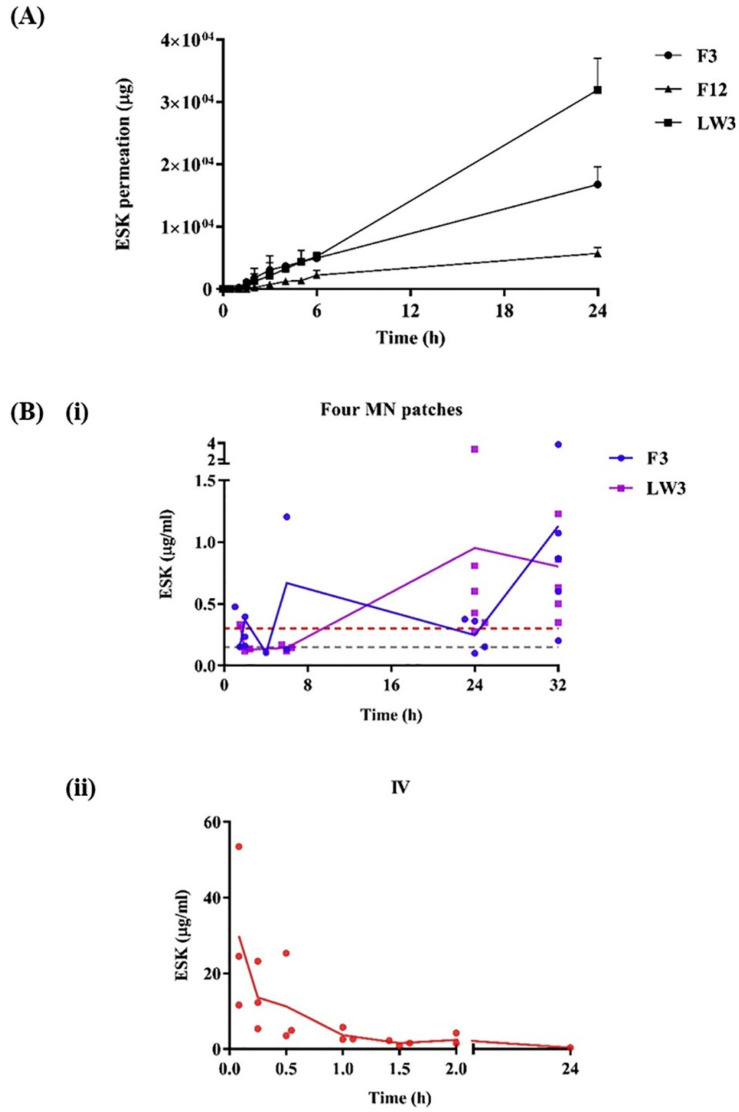
(**A**) In vitro permeation profiles of ESK from MN patches consisting of lead formulations in combination with hydrogel-forming MN arrays (means + S.D., n = 4). (**B**) ESK concentration in rat plasma. (**i**) Treatment cohorts that received 4 MN patches consisting of either a film (formulation code, F3) or lyophilized wafer (formulation code, LW3) as the drug-containing reservoir (F3, means ± S.D., n = 3 at 1.5 h, 2 h, 4 h, and 6 h; n = 4 at 24 h; n = 6 at 32 h) (LW3, means ± S.D., n = 3 at 1.5 h, 2 h, and 6 h; n = 6 at 24 h and 32 h). Red and grey dashed lines indicate the target plasma concentrations, 0.15 μg/mL and 0.3 μg/mL, respectively. Purple and blue solid lines indicate the average ESK concentration in rat plasma. (**ii**) Control cohort that received ESK solution via IV (means ± S.D., n = 3). The red solid line indicates the average ESK concentration in rat plasma [28].

Concurrently, new methods for fabricating aqueous hydrogel-forming microneedle arrays have emerged, utilizing an innovative, scalable injection molding technique. This approach employs metal master templates to create silicone molds, representing a substantial improvement over the older centrifugation method. Testing has validated the functionality of these microneedles, showing successful skin insertion with minimal structural damage [6]. In another innovative development, hydrogel-forming microneedle arrays containing esketamine (ESK) demonstrated efficacy in drug delivery. Utilizing polymeric films and lyophilized reservoirs, these arrays have shown promising results in enhancing plasma concentrations and sustaining drug release in rat models. This progress suggests that these microneedle arrays could replace intravenous administration of esketamine, thereby improving patient compliance and treatment accessibility [28]. Figure 1 illustrates the in vitro permeation of ESK from MN patches and in vivo delivery of ESK from Sprague Dawley rats.

Further advancements have been made with hydrogel-forming microneedles incorporating a poly(ethylene glycol) reservoir. A system using polyvinyl alcohol and polyvinylpyrrolidone crosslinked with tartaric acid has proven effective in transdermally delivering sildenafil citrate, thereby improving therapy for erectile dysfunction by enhancing bioavailability and patient compliance [35].

Parallel developments in microneedle systems include the transdermal delivery of albendazole for treating cystic echinococcosis. This system also incorporates a poly(ethylene glycol) reservoir with microneedles made from polyvinyl alcohol and polyvinylpyrrolidone crosslinked with citric acid. It has significantly enhanced drug permeation while preserving skin integrity, increasing bioavailability, and circumventing first-pass metabolism without causing irritation or pain [36].

Advancements in microneedle technology have also been applied to the treatment of pulmonary hypertension. A novel hydrogel-forming microneedle patch, made from polyvinyl alcohol and polyvinylpyrrolidone crosslinked with citric acid, includes a direct-compressed tablet reservoir of sildenafil citrate. This patch, designed to improve drug solubility and patient comfort, has demonstrated robust insertion capabilities and effective drug release in ex vivo studies. These features enhance therapeutic efficacy for pulmonary hypertension by facilitating steady, prolonged drug delivery directly through the skin [15].

Another innovative hydrogel-forming microneedle array consists of polyvinyl alcohol and polyvinylpyrrolidone crosslinked with citric acid, incorporating methotrexate in reservoirs made from hydroxypropyl methylcellulose and glycerol. Developed for the sustained transdermal delivery of methotrexate, this patch has shown promising results in preclinical studies, offering improved plasma levels and reduced side effects. This approach effectively overcomes skin barrier limitations, providing sustained drug delivery with minimal irritation [16].

The field of microneedle technology continues to advance with the development of a self-adhesive microneedle patch made from methacrylated hyaluronic acid. This patch can load and release various therapeutics through a swelling effect, positioning it as a versatile platform for transdermal drug delivery. This swellable hydrogel microneedle patch has been investigated for its enhanced drug load and adhesion properties, showing broad applicability in improving therapeutic outcomes across different drug types [37].

Further innovations include the integration of 2-hydroxyethyl methacrylate (HEMA) and ethylene glycol dimethacrylate (EGDMA), loaded with 5% (*w*/*w*) ibuprofen, into light-responsive 3,5-dimethoxybenzoin conjugates. This represents novel light-responsive hydrogel-forming MNAs that enable controlled, on-demand drug release activated by light exposure. This technology allows for prolonged and controlled drug delivery mediated by optical signals [9].

Moreover, directly compressed tablets (DCTs) integrated with polyvinyl alcohol-based hydrogel-forming MN arrays have been developed for delivering drugs, such as amoxicillin, levodopa/carbidopa, and levofloxacin. These non-aqueous-based DCTs within MN patches are specifically designed for drugs unstable in aqueous environments, with in vivo studies validating their effective transdermal delivery. This strategy broadens the range of drugs that can be transdermally administered while addressing stability issues [38].

A formulation composed of poly(ethylene glycol) (10,000 Da), poly(methyl vinyl ether/maleic anhydride) copolymer (1,980,000 Da), and sodium carbonate (3% *w*/*w*) in a ratio of 1:3 (7.5%:22.5% *w*/*w*) has been developed. This specific composition has markedly enhanced the transdermal delivery of acyclovir, achieving a 39-fold increase in skin permeation compared to the control, with sustained release over 24 h. This indicates its potential for daily application, improving drug bioavailability and patient compliance [39].

Additionally, super-swelling hydrogel-forming microneedles (HFMNs) crafted from aqueous blends of Gantrez^®^ S-97, poly(ethylene glycol), and anhydrous sodium carbonate (Na_2_CO_3_) have been extensively studied for their swelling ratios and drug transport capabilities. Mathematical modeling, including drug binding within the matrix, was developed to simulate drug transport through these HFMNs. In vitro diffusion studies using ibuprofen sodium across porcine skin confirmed that enhanced drug solubility in the reservoir leads to increased drug transport across the skin, thereby boosting the efficacy of drug delivery systems [40].

The development of MNAs has also seen remarkable innovations with the introduction of a rigid, resin-based outer layer, 3D-printed onto a conformal backing filled with drug-eluting hydrogels. These microneedles, varying in length, are designed specifically for the delivery of Vascular Endothelial Growth Factor (VEGF). This approach leverages the encapsulation capabilities of hydrogels with the deep penetration power of microneedles, addressing the mechanical challenges posed by hydrogel use alone. By tailoring the needle geometry and hydrogel composition, these MNAs provide controlled spatial and temporal delivery of therapeutics [41].

Additionally, polyvinyl alcohol (PVA)-based hydrogel microneedle patches have been developed, featuring variations in saponification degrees from PVA6 to PVA10. These patches are engineered to have adjustable disassembly rates while maintaining morphological stability and efficient penetration. Their non-toxic nature and high cell viability—about 80%—have been confirmed, validating their suitability for human use and demonstrating the versatility of saponification ratios in customizing disassembly properties [18].

Table 1 provides an extensive evaluation of swellable microneedle systems designed to improve transdermal drug delivery. Each entry outlines the study aim and the microneedle structure. The second column integrates mixed study findings with the authors’ critical assessment, highlighting limitations, and complementary studies for each system from hydrogel-forming MNAs for delivering different therapeutic agents to light-responsive MNAs. These innovations offer promising solutions for controlled drug release and improved patient compliance. However, challenges, such as manufacturing complexity and potential skin reactions, highlight the need for further investigation and optimization in real-world applications.

## 3. Potential Applications 

### 3.1. Swellable Microneedles in Insulin Delivery

Recent advancements in microneedle technology have significantly improved the efficacy and accessibility of insulin delivery systems for diabetes care. One notable innovation is the porous reservoir-backed microneedle patch made from polyvinyl alcohol, featuring a boronated smart gel tip and a crystallized surface coating. This design enhances skin penetration and mechanical robustness, enabling prolonged, glucose-responsive insulin delivery. This advancement has been shown to increase treatment efficiency and patient adherence, marking a significant step forward in glucose management for diabetes patients [42].

Additionally, a thermally stable microneedle patch has been developed using a boronate-gel-based, glucose-responsive hydrogel. This patch maintains a consistent rate of insulin release regardless of temperature variations and independent of glucose oxidase or nanoparticles. This feature makes it an excellent candidate for synthetic, large-scale production, promoting sustainable manufacturing and supporting autonomous insulin delivery [12].

Further innovations include a microneedle patch produced through the copolymerization of N-isopropyl acrylamide, N-vinyl-2-pyrrolidone, and 3-acrylamidophenylboronic acid, with insulin incorporated directly via a mild drop/dry method. This technique allows for glucose-dependent release of insulin, optimally tuned to physiological conditions, offering a viable method for incorporating insulin into polymerized, glucose-responsive microneedles [43].

Another advancement features a microneedle patch composed of a hydrogel made from phenylboronic-acid-grafted polyallylamine and polyvinyl alcohol, crosslinked via boronate ester bonds. This design enables modifications in crosslink density in response to varying glucose levels, simplifying the manufacturing process while ensuring precise control of insulin release. The efficacy of this patch has been validated in diabetic rat models, demonstrating its potential for simplified production and improved insulin delivery management [44].

Moreover, a microneedle patch integrating a hydrogel composed of phenylboronic-acid-grafted sodium hyaluronate and polyvinyl alcohol, reinforced with cellulose nanofibers for enhanced mechanical strength, has been developed. This patch combines ease of manufacturing with high insulin-loading capabilities, responding adeptly to glucose fluctuations through reversible boronate ester bonding. Its straightforward production process and substantial loading capacity make it an ideal choice for mass production, enhancing the practicality of this innovative delivery system [45].

In a similar domain, a painless core–shell microneedle array has been designed, featuring a crosslinked gel responsive to hydrogen peroxide and embedded with glucose-specific and hydrogen-peroxide-scavenging enzymes. This glucose-responsive system effectively controls blood glucose levels in diabetic mouse models, offering rapid response and exceptional biocompatibility. It represents a significant improvement in the treatment of both type 1 and advanced type 2 diabetes, enabling precise insulin release tailored to fluctuating glucose levels [14].

### 3.2. Swellable Microneedles in Psoriasis Management

Recent advances in microneedle technology have opened up new treatment options for chronic conditions, such as psoriasis. A notable innovation is the development of detachable gel-based microneedle patches that are responsive to hydrogen peroxide. These patches contain methotrexate and epigallocatechin gallate (EGCG), utilizing EGCG’s dual role as both a crosslinker and an anti-inflammatory agent. This combination not only ensures prolonged retention on the skin but also enhances the treatment’s effectiveness in animal studies. The patches feature a dual-mode drug release system and intelligently respond to reactive oxygen species (ROS), providing sustained management of psoriasis [10].

**Figure 2:**
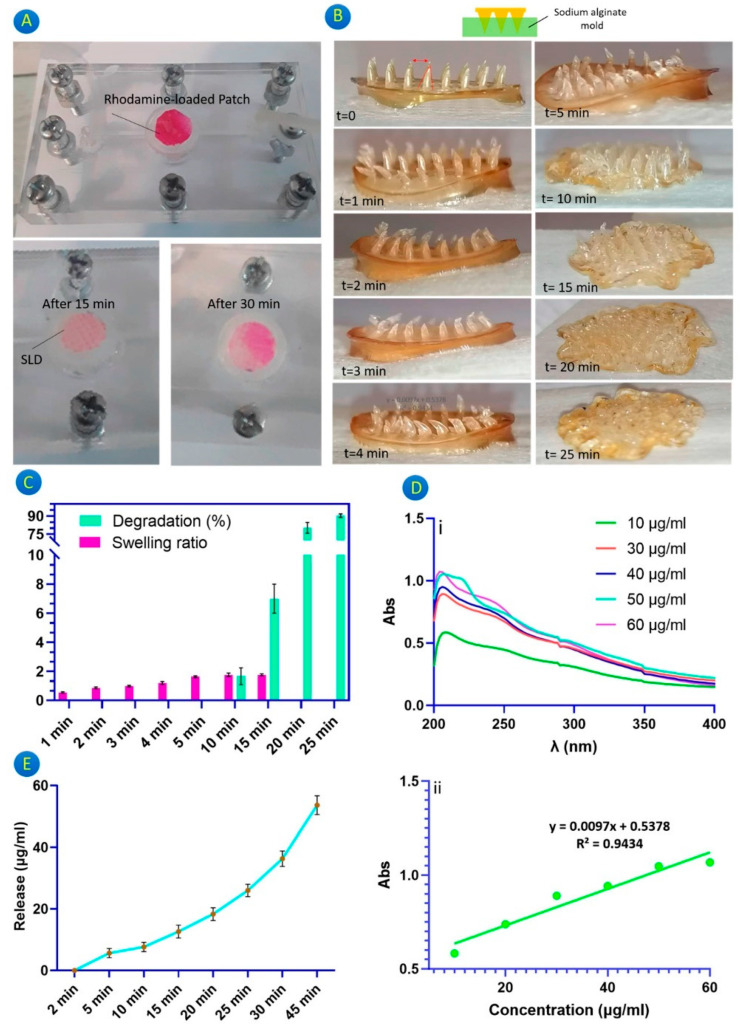
(**A**) Rhodamine release in SLD matrix evaluation using a microfluidic system, (**B**) visual approach to monitor swelling of microneedles, (**C**) swelling and degradation behavior of the GgE-loaded MNP, (**D**) standard curve to determine the exact concentration of released GgE, and (**E**) release profile of GgE from GgE-loaded MNP [13].

Another development is the creation of a chitosan-based microneedle patch designed for delivering Glycyrrhiza glabra extract in dermatological applications. Fabricated using a CO_2_ laser cutter, this patch offers a cost-effective treatment option for psoriasis. It facilitates a burst release of the active ingredient, with the microneedles showing substantial swelling and rapid degradability in vitro. These characteristics are vital for quickly alleviating acute psoriasis symptoms, demonstrating the patch’s therapeutic potential [13]. Figure 2 illustrates the rhodamine release via a microfluidic system, the swelling and degradation behavior of the microneedles, the behavior of a gallic-acid-loaded microneedle patch, and the release profile of gallic acid.

Further advancements in microneedle technology include a patch featuring a black-phosphorus-loaded hydrogel made from N-isopropyl acrylamide and poly(ethylene glycol) diacrylate, with an inverse opal structure filled with gelatin/agarose. This sophisticated design targets psoriasis treatment and integrated photothermal responsiveness. It also enables real-time monitoring of drug delivery through changes in structural color. This microneedle patch combines excellent material properties and structural innovation, facilitating effective treatment of skin diseases [46].

Additionally, a tip-swellable microneedle array has been developed using photo-crosslinked methacrylated hyaluronic acid and a biocompatible resin for delivering methotrexate. This approach enhances the efficiency of drug delivery and therapeutic outcomes in psoriasis treatment. By offering a user-friendly and efficient alternative for administering methotrexate, this design reduces side effects and improves drug retention at the target site. Such advancements could significantly decrease the overall treatment burden for patients [47].

### 3.3. Swellable Microneedles in Pain and Arthritis Management

Hydrogel-based microneedle devices have emerged as a promising innovation in pain management. Incorporating transdermal electro-modulation, these devices have undergone extensive testing both in vitro and in vivo using a rat model to evaluate their effectiveness in delivering analgesia. The results have demonstrated promising pharmacokinetic profiles and significant analgesic effects. The strong positive correlation observed between the in vitro and in vivo outcomes underscores the substantial potential of these microneedles for clinical applications in pain management, offering a novel method for pain relief administration [48].

In arthritis treatment, hydrogel microneedles have been designed to enhance stability and efficacy. These microneedles are loaded with a DEK-targeting aptamer and feature specific modifications, including methoxy groups on each deoxyribose sugar unit, inverted deoxythymidine at the 3′ end, and cholesterol at the 5′ end. The aptamer’s improved stability and bioavailability facilitate effective transdermal delivery, significantly reducing inflammation and joint damage in mice [49].

Further advancements in hydrogel microneedle technology include the development of a UV-responsive crosslinker, resulting in a durable, super-swelling structure capable of delivering colchicine for the acute treatment of gout arthritis. These highly swellable microneedles have demonstrated their effectiveness by delivering anti-inflammatory drugs and showing significant therapeutic effects in vivo, thus improving the management of acute gout arthritis [50].

### 3.4. Swellable Microneedles in Specific Therapeutic Areas

In cardiovascular therapy, a microneedle patch made of gelatin methacryloyl (GelMA) loaded with galunisertib, a TGF-beta inhibitor, has shown promising results for cardiac repair following myocardial infarction. This patch provides sustained drug release for more than two weeks and improves cardiac function by inhibiting fibrosis, offering a targeted antifibrotic delivery method directly to the affected area [51].

Similarly, another GelMA-based hydrogel microneedle patch has been developed for the sustained release of pirfenidone, aimed at treating liver fibrosis. Demonstrated in a mouse model, this patch has shown good biocompatibility and enhanced therapeutic efficacy, providing an efficient treatment option for critically ill patients who cannot take oral medications [52].

For spinal cord injury therapy, a novel three-dimensional-cultured mesenchymal stem cell (MSC)-derived exosome–hydrogel hybrid microneedle array patch has been introduced. This innovative delivery method significantly reduces inflammation and glial scarring, enhancing the therapeutic potential of MSC-derived exosomes in spinal cord injury treatment [53].

Additionally, a photothermal microneedle hydrogel patch has been introduced for treating soft tissue injuries. This patch contains taurine-loaded Prussian blue nanoparticles encapsulated in a methacrylate-based hyaluronic acid hydrogel. Its photothermal properties promote healing through thermo-sensitized anti-inflammatory mechanisms, offering an innovative method for treating soft tissue injuries with effective healing and minimized systemic side effects [54].

In conclusion, swellable microneedles represent a significant advancement in the field of pain management and various therapeutic areas. Their ability to deliver drugs efficiently, with improved stability and targeted release, highlights their potential for enhancing patient outcomes across multiple medical conditions.

Table 2 provides a comparative assessment of hydrogel-based microneedle systems designed for responsive drug delivery. The second column integrates mixed study findings alongside the authors’ critical evaluation, elucidating limitations, and complementary studies of each system. From glucose-responsive insulin delivery to treatments for various conditions, such as psoriasis, arthritis, and spinal cord injury, this overview offers insights into the potential applications and challenges associated with these innovative drug delivery platforms.

## 4. Biomedical Applications, Sensing, and Monitoring

Given the severity and prevalence of cancer, innovative strategies for treatment and diagnostics are essential. Theranostic hydrogels offer a personalized approach that combines multiple functions on a single platform, enhancing patient comfort and facilitating clinical translation. Nanoparticles are widely used in theranostic systems for their ability to circulate systemically, evade host defenses, and deliver drugs precisely at the target site. Combining nanoparticles with hydrogel microneedles enhances their theranostic potential [55]. This section reviews recent advances in hydrogel microneedles as theranostic platforms, focusing on cancer therapy, glucose monitoring, therapeutic drug monitoring, and other disease conditions.

### 4.1. Biomarker Sampling and Disease Diagnosis

Recent advancements in microneedle technology have significantly expanded their diagnostic applications, particularly in the field of sensing and sampling interstitial fluids for health monitoring. A notable innovation in this field is the development of microneedles coated with an alginate–peptide nucleic acid hybrid. These microneedles facilitate minimally invasive sampling and specific sensing of nucleic acids from the skin’s interstitial fluid, demonstrating rapid sampling and automated detection capabilities. This technology shows great promise for early disease diagnosis and continuous health monitoring [29].

Another development involves the use of crosslinked poly(ethylene glycol) diacrylate (PEGDA) MNAs designed for the extraction of interstitial fluid. These MNAs are integrated with colorimetric sensors for detecting biomarkers. The 3D-printed microneedle arrays not only facilitate the extraction of interstitial fluid but also enable real-time colorimetric detection of biomarkers. Their design, which has been thoroughly evaluated for needle shapes and mechanical properties, paves the way for quick and efficient home-based monitoring of metabolic diseases using a self-administered device [56].

**Figure 3:**
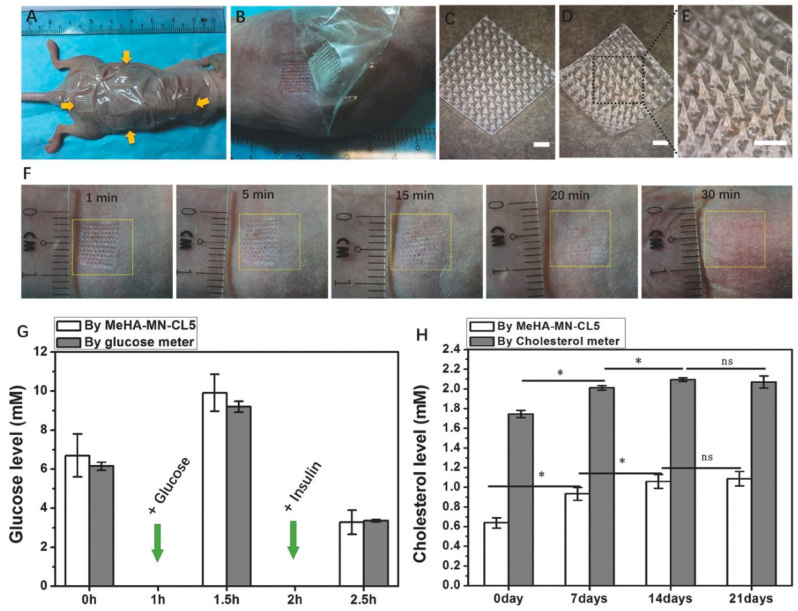
The extraction of skin ISF and subsequent analysis of metabolites (glucose and cholesterol) by using the MeHA-MN patches with 5 min of UV exposure (namely, MeHA-MN-CL5) in the mouse model. (**A**) Four MN patches were thumb-pressed into the skin of the mouse’s back and then fixed using 3M Tegaderm film. (**B**) The microholes caused by the penetration of the MN patch. The image of the MN patch (**C**) before penetration and (**D**) after the removal from mouse skin. (**E**) Zooming visualization of the MN patch after the removal from the mouse skin. (**F**) The skin recovery after the treatment. The microholes made by the MN patch gradually disappeared within 30 min. (**G**) The comparison of the glucose level in the mice detected with the commercial glucometer and the colorimetric method aided by the MN patch. The intraperitoneal injections of glucose (1 g kg^−1^) and insulin (2 U kg^−1^) were applied to regulate the glucose level. After injection, it needs 30 min to avoid lag time before the ISF extraction and subsequent glucose quantification. (**H**) Comparison of the cholesterol level in the mice detected using a commercial cholesterol meter and the colorimetric method aided by the MN patch. The mice were fed a high-fat diet for three weeks before the experiment. Scale bar: 1000 µm in (**C**–**F**). * *p* < 0.05, ns: no significant difference [23].

Moreover, a swellable microneedle patch composed of methacrylated hyaluronic acid (MeHA) crosslinked through UV irradiation has been developed for the rapid extraction of skin interstitial fluid. This patch enhances the ability to perform timely metabolic analyses without the need for additional device assistance while maintaining structural integrity during use. It offers a straightforward method for metabolic monitoring and diagnosis, leveraging the simplicity and efficiency of microneedle technology [23]. Figure 3 illustrates the in vivo performance of the MeHA-MN patch in a mouse model.

In oncology, a swellable microneedle made of polyvinyl alcohol (PVA) and chitosan (CS) has been developed to extract skin interstitial fluid and visually quantify the biomarker S100A1 using a microfluidic particle dam. This microneedle system, tailored for on-site melanoma diagnosis, facilitates the extraction and quantification of S100A1 directly from interstitial fluid, showing high sensitivity compared to traditional serum-based methods. This approach achieves precise melanoma diagnosis with a low detection limit and robust visual feedback, providing a significant tool for the early detection and management of melanoma [30].

An innovative approach features a swellable hydrogel microneedle composed of polyvinyl alcohol and sodium alginate, integrated with a cerium–metal organic framework composite nanozyme for detecting critical biomarkers, such as glutathione. This microneedle-based system offers rapid and minimally invasive detection of biomarkers in interstitial fluid, showing significant promise for in vivo applications and potentially enhancing diagnostic procedures considerably [24].

Additionally, a swellable bilateral core–shell microneedle patch has been developed, featuring a shell layer for rapid interstitial fluid absorption and a core layer that reacts with lactate to provide colorimetric analysis of lactate levels directly from the skin. This microneedle patch is designed for rapid and accurate in situ analysis of lactate levels, proving particularly useful in sports medicine and the early diagnosis of melanoma. Comparative studies with blood lactate levels during exercise and in melanoma models have indicated that this patch can successfully measure changes in lactate in real time, with a high correlation to blood levels. It has also demonstrated potential in detecting early-stage melanoma in animal models [25].

### 4.2. Real-Time Monitoring and Sensor Integration

Real-time monitoring and sensor integration have seen significant advancements with the introduction of innovative microneedle technologies. One notable development is the creation of a conductive hydrogel microneedle array incorporating poly(3,4-ethylenedioxythiophene) polystyrene sulfonate (PEDOT:PSS) and silver–platinum nanoparticles. This system represents a major breakthrough in enzyme-less electrochemical glucose sensing. When assessed in a diabetes model, this continuous glucose monitoring (CGM) system demonstrated enhanced performance, suggesting its potential for wider applications in disease management [19].

Moreover, a microneedle array made from dopamine-conjugated hyaluronic acid embedded with PEDOT:PSS has been developed for real-time pH measurement in live animals. This innovative system bridges biosensing with interstitial fluid extraction, allowing continuous health monitoring directly on the needle. This on-needle pH detection could expand the scope of biosensing applications and foster new strategies for continuous health monitoring [26].

**Figure 4:**
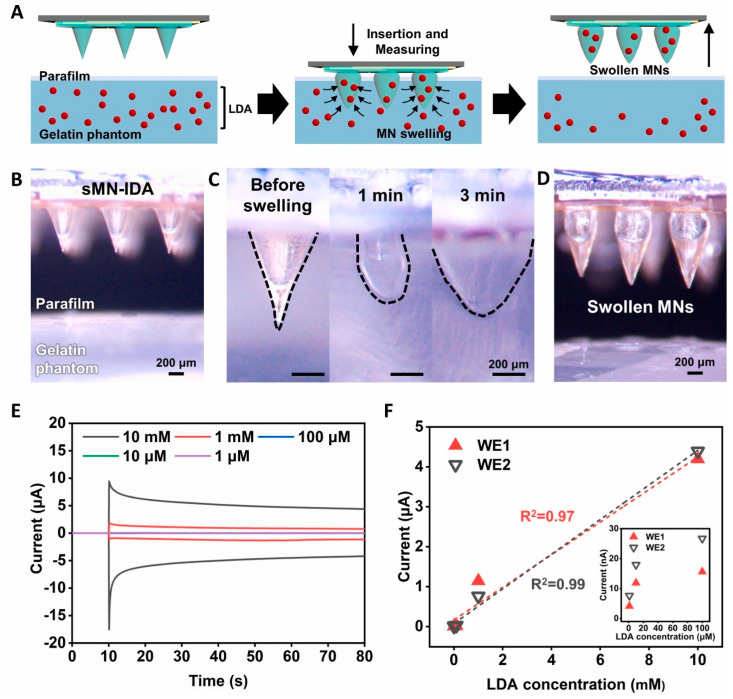
Analysis of LDA in the model phantom. (**A**) Schematic images of the in vitro measurement test using the sMN-IDA sensor and the gelatin phantom, which contained various concentrations of LDA. The sMN-IDA sensor was inserted into the gelatin phantom and measured after 3 min of swelling. (**B**) Stereo microscopic images of the sMN-IDA sensor before insertion. (**C**) Swelling behavior of a sMN in the gelatin phantom in 3 min. (**D**) Stereo microscopic images of the swollen sMN-IDA sensor after the measurement. (**E**) The amplified redox cycling current was measured with various LDA concentrations from 10 mM down to 1 μM. (**F**) Redox currents of each WE in 80 s of LDA with a concentration from 1 μM to 10 mM in 10-fold increments. Inset plot represents the redox currents at 80 s of LDA with a concentration from 1 μM to 100 μM in 10-fold increments [57].

Further advancements include the development of a swellable microneedle-mounted nanogap sensor designed for single-step sensing of levodopa. This sensor is characterized by rapid absorption of interstitial fluid and enhanced signal amplification through redox cycling within the nanogap electrodes. Its high sensitivity and specificity enable in situ measurement of levodopa in skin tissue, offering significant benefits for self-diagnosis in certain conditions, such as Parkinson’s disease, and improving the accuracy and convenience of patient-managed care [57]. Figure 4 illustrates the levodopa measurements in a model phantom, highlighting the potential for monitoring the target species within human tissue.

In another development, swellable hydrogel microneedles composed of polyvinyl alcohol and polyvinylpyrrolidone (PVA/PVP) have been designed for the rapid extraction of interstitial fluid to monitor glucose levels. This method has been evaluated for efficiency and accuracy, showing promise in non-invasive disease monitoring and potentially enhancing clinical disease prevention and diagnosis [58].

A wearable transdermal microneedle patch has also been created, integrating a photonic crystal hydrogel functionalized with phenylboronic acid for in situ glucose monitoring. This minimally invasive system uses a colorimetric response visible to the naked eye, simplifying diabetes management and potentially extending to the detection of other biomarkers in clinical settings [20].

Another advancement is a nanometallic conductive composite–hydrogel core–shell microneedle skin patch designed specifically for glucose monitoring. This system features a conductive composite core and hydrogel shell, along with signal processing units, facilitating real-time data analysis and marking a significant step forward in wearable devices for continuous, non-invasive health monitoring [21].

Microneedles equipped with aptamer probes have been introduced for the fluorescence detection of biomarkers, such as glucose and thrombin, without the need for additional reagents. This on-needle fluorescence detection method enables rapid and sensitive in vivo monitoring, particularly in diabetes models, emphasizing its potential for point-of-care testing by eliminating the need for external sample processing [59].

Furthermore, microneedles composed of hydrolyzed poly(methyl-vinyl ether-co-maleic anhydride) and poly(ethylene glycol) have shown promising capabilities for in vivo detection of substances like caffeine and glucose. These findings offer strong proof of concept for the use of hydrogel-forming microneedles in therapeutic drug monitoring and minimally invasive patient monitoring [22].

### 4.3. Minimally Invasive Sampling and Quantification

Minimally invasive sampling and quantification using swellable microneedles is a rapidly advancing field, particularly due to the development of hydrogel-forming microneedle arrays. Arrays composed of hydrolyzed poly(methyl-vinyl ether-co-maleic anhydride) crosslinked with poly(ethylene glycol) have shown promising results in outpatient settings for lithium monitoring. Such microneedles enable dose-dependent extraction of lithium, offering significant potential for monitoring lithium levels in patients with psychiatric conditions, thus facilitating effective disease management [60].

Moreover, hydrogel microneedle patches made from polyvinyl alcohol (PVA) and chitosan (CS) have been optimized for efficient interstitial fluid extraction and rapid biomarker recovery due to their highly porous structure. These patches support point-of-care testing (POCT), as demonstrated by their application in glucose level monitoring in rabbits. Their design makes them highly suitable for rapid biomarker recovery and immediate clinical application, enhancing their applicability in POCT scenarios [61].

The diagnostic landscape is further enriched by innovative microneedle-based systems that significantly enhance patient monitoring and disease diagnosis. A noteworthy development is the controllable swelling microneedle patch made from poly(ethylene glycol) diacrylate and methacrylated hyaluronic acid. This patch enables the recovery of analytes onto moist cellulose paper, which can be detected via enzymatic colorimetric reagents or plasmonic arrays. Initially applied to nicotine monitoring in humans, this microneedle-assisted paper-based sensing platform provides rapid, painless biofluid analysis with ultrasensitive molecular recognition, greatly improving personal health monitoring capabilities [62].

Further advancements in hydrogel microneedle technology include arrays integrated with a graphene oxide–nucleic acid (GO.NA)-based fluorescence biosensor for in situ detection of biomarkers, such as glucose and uric acid. This assay combines a nucleic acid probe with a portable detector, enabling effective measurement of multiple biomarkers both ex vivo and in vivo. The integration of minimally invasive sampling with advanced sensing techniques highlights the potential of this assay for comprehensive POCT, thereby enhancing diagnostic efficiency [63].

## 5. Miscellaneous Applications

### 5.1. Scar Management and Treatment

Innovative microneedle technologies have significantly advanced regenerative medicine and wound healing. One notable development is a hydrogel microneedle patch composed of methacrylate gelatin (GelMA) and poly(ethylene glycol) diacrylate (PEGDA), loaded with compound betamethasone (CB). This patch provides a less painful method for the intradermal delivery of corticosteroids, targeting hypertrophic scars specifically. Demonstrated in rabbit models, it ensures efficient intradermal delivery and sustained drug release, significantly reducing scar tissue formation [64]. Another approach involves a hydrogel-forming adhesive DL-MN patch, which achieves wound closure and healing in vivo. Additionally, an antimicrobial hydrogel microneedle system incorporating bismuth nanosheets (Bi) and verteporfin (Vp) has been developed. This system effectively blocks mechanotransduction signaling, promoting scarless healing. The detachable system facilitates continuous drug release, inhibits fibrosis, and aids skin regeneration by suppressing YAP signaling, offering a novel approach to scar-free regenerative treatments [65].

### 5.2. Wound Healing (Diabetic and Chronic Wounds)

An advanced hydrogel composed of gelatin methacrylate (GelMA), 4-(2-acrylamidoethylcarbamoyl)-3-fluorophenylboronic acid (AFPBA), and gluconic insulin (G-insulin) has been developed for glucose-responsive insulin delivery. This innovative hydrogel microneedle dressing enhances diabetic wound healing by regulating insulin release and improving recovery in animal models. It exhibits high biocompatibility and dynamic insulin release in response to changing glucose levels, thus facilitating the healing process [66]. Moreover, a novel bacterial-responsive microneedle dressing featuring a hydrogel backing layer with doxycycline hydrochloride-loaded polycaprolactone microspheres has been created. This dual-function dressing disrupts biofilms through microneedle-mediated mechanisms and provides sustained therapeutic agent delivery via its hydrogel layer. This method significantly advances the healing of chronic wounds by integrating mechanical debridement with therapeutic delivery, offering an effective strategy for infection management and chronic wound healing [67].

Furthermore, a novel oxygen-producing silk fibroin methacryloyl hydrogel microneedle patch has been created. This patch features tips encapsulated with calcium peroxide and catalase, releasing oxygen continuously and inhibiting reactive oxygen species, while the base coated with Ag nanoparticles combats infections. This multifunctional patch accelerates diabetic wound healing by promoting cellular processes and reducing infections, making it a promising clinical strategy [68].

### 5.3. Surgical Applications and Infection Control

**Figure 5:**
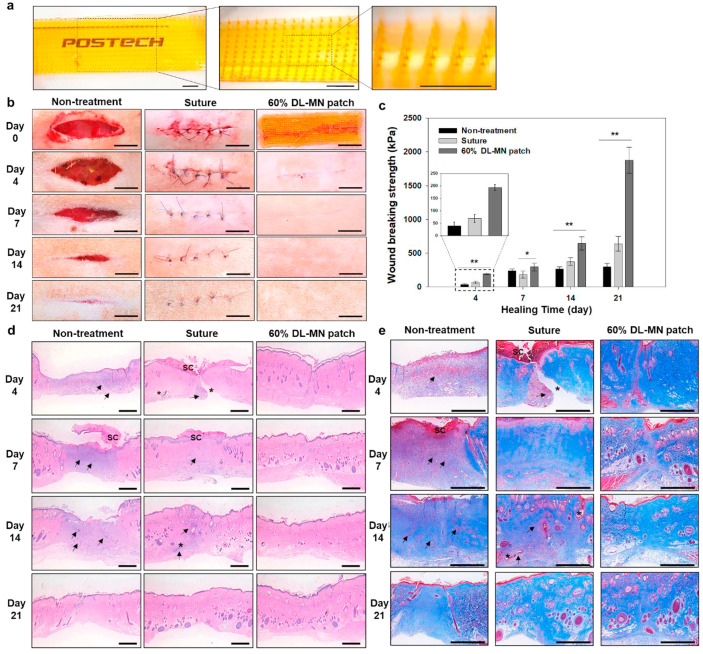
In vivo external wound closure and healing. (**a**) Images of the transparent strip-type 60% DL-MN patch. Scale bar = 2 mm. (**b**) Macroscopic images for the healing of rat skin wounds after non-treatment, suture treatment, and 60% DL-MN patch treatment during the observation period. Scale bar = 1 cm. (**c**) Ultimate breaking strength of wounds after non-treatment, suture treatment, and 60% DL-MN patch treatment. A magnification box shows the tensile strength at 4 days post-wounding. The results from at least 5 samples were averaged to obtain each value. Statistical significance is designated as follows: ** *p* < 0.01 and * *p* < 0.05. Histological observations of (**d**) H&E− and (**e**) MT-stained skin wounds after non-treatment, suture treatment, and 60% DL-MN patch treatment. Scabs (sc), inflammation (black arrow), and defects (black star) are indicated. Scale bar = 500 μm [69].

A bio-inspired, double-layered adhesive microneedle patch has been introduced, combining a swellable mussel adhesive protein (MAP)-based shell with a non-swellable silk fibroin (SF)-based core. This patch demonstrates superior sealing capabilities on dynamic tissues, proving effective both ex vivo and in vivo for sealing internal and external wounds. This innovative technology has the potential to replace traditional sutures and staples, offering a less invasive and more efficient alternative for wound closure [69]. Figure 5 illustrates the in vivo external wound closure and healing using the hydrogel-forming adhesive MN patch.

Furthermore, MgO@polydopamine (MgO@PDA) nanoparticle-loaded photothermal microneedle patches, used in conjunction with chitosan (CS) gel dressings, have been developed for treating infectious wounds. This combined approach enhances drug delivery depth, increases load capacity, and synergizes photothermal effects under near-infrared irradiation, improving drug delivery, antibacterial activity, and wound healing [70].

### 5.4. Immunotherapy and Other Innovations

A study using ovalbumin as a model protein antigen evaluated the immunogenicity and efficacy of novel hydrogel-forming microneedles compared to traditional dissolving microneedles for vaccine delivery. The research revealed significant differences in immune responses elicited by each microneedle type, underscoring the impact of microneedle design and the interactions between the polymer and the antigen on vaccine immunogenicity. This study emphasizes the critical role of microneedle design in enhancing vaccine efficacy and provides insights into potential optimizations of microneedle configurations for improved immune responses [71]. Additionally, a functional microarray system combined with a hydrogel patch containing hepatitis B surface antigen (HBsAg) and cholera toxin B (CTB) as an adjuvant has been assessed for transcutaneous immunization. This innovative strategy aims to enhance antigen permeation through the skin and induce stronger immune responses compared to conventional methods. The results showed significantly increased antigen permeation and stability, along with enhanced immunogenicity, demonstrating the potential of advanced microneedle systems in improving vaccine delivery and efficacy through non-invasive transcutaneous immunization approaches [72].

Further innovations in the development of agarose gel microneedles have emerged. Using a photothermal method with a 1064 nm laser focused on a photoabsorbent chromium layer, these microneedles are etched to facilitate the development of 3D microfabricated environments. This advancement is particularly important for isolated cell spheroid growth, which is crucial for studying community effects during drug screening. These innovations enhance the precision of cellular microenvironments and provide valuable insights into multicellular interactions within these settings [17].

### 5.5. Treating Alopecia

Alopecia Areata is characterized by localized hair loss due to autoimmunity, traditionally managed with systemic immunosuppression, which risks infections and malignancies. To address this, a hydrogel microneedle (MN) patch delivering CCL22 (a T(reg) chemoattractant) and IL-2 (a T(reg) survival factor) was developed to locally expand regulatory T cells (T(regs)). This approach restored immune balance and promoted sustained hair regrowth in a murine model without peripheral immunosuppression in a humanized skin transplant mouse model. The MN patch demonstrated high-loading capacity and shelf-life stability, highlighting its potential for clinical use in autoimmune skin disease management [73].

Androgenetic Alopecia (AGA) is characterized by hair loss due to downregulated angiogenic genes and poor vascularization. A novel hyaluronic acid (HA)-based hydrogel microneedle (MN) patch, V-R-MNs, was designed to deliver Vascular Endothelial Growth Factor (VEGF) and Ritlecitinib, encapsulated in polyhydroxyalkanoate (PHA) nanoparticles. This minimally invasive treatment improved skin penetration, angiogenesis, and the immune microenvironment around hair follicles, promoting hair follicle cell proliferation and development. In AGA model mice, V-R-MNs outperformed the clinical drug minoxidil, showing rapid anagen phase onset, improved hair quality, and greater coverage. This presents a safer and more effective strategy for AGA treatment, with potential applications for other related diseases [74].

Table 3 offers a comparative analysis of hydrogel microneedle systems designed for biomarker detection, monitoring, wound healing, and tissue engineering. The second column synthesizes mixed study findings with the authors’ critical assessment, limitations, and complementary studies. From glucose monitoring to drug detection, scar treatment, diabetic wound healing, and alopecia treatment, these systems aim to enable minimally invasive point-of-care testing. However, challenges, such as sensor stability, reliability, precise dosing, long-term effects, manufacturing complexity, and clinical validation persist across various applications.

## 6. User Experience and Safety

Recent studies have focused on the self-application of hydrogel-forming microneedle array patches (MAPs) by patients, supported by pharmacists and detailed patient information leaflets. Research involving human volunteers has demonstrated that these microneedle arrays can effectively penetrate the skin and perform their intended functions, highlighting the practicality and efficacy of patient-led approaches. This method enhances patient compliance and autonomy in managing their treatment protocols, promoting a more patient-centered approach to healthcare [7].

Extensive evaluations of the safety of hydrogel-forming microneedle arrays, particularly regarding their repeated use, have been conducted. Investigations into skin reactions and systemic biomarker concentrations have confirmed their safety for repeated applications in humans. These findings support their use in clinical settings for continuous patient monitoring and treatment, reinforcing both their safety profile and clinical utility [27].

In addition to safety, the effectiveness of microneedle insertion during self-application has been enhanced by integrating a pressure-indicating sensor film (PISF) with these arrays. Results have shown that the sensor film reliably indicates correct insertion, maintaining consistent penetration depths and ensuring functional performance. This technology provides immediate feedback to users, enhancing confidence and potentially improving clinical outcomes, thereby making microneedle arrays more user-friendly [8].

Another innovation is the integration of a hydrogel-forming microneedle array with a water-filled reservoir that provides audible and tactile feedback upon successful skin insertion. This feature ensures effective skin penetration without compromising drug delivery, facilitating accurate and reliable self-application. Such advancements are crucial for clinical reliability and patient adherence [75].

Ongoing research has also addressed concerns regarding microbial penetration and storage stability, which are essential for ensuring safety and efficacy in clinical environments. Studies have confirmed the antimicrobial properties and storage stability of these microneedle arrays, establishing a solid foundation for further clinical development and setting safety standards for transdermal drug delivery systems [76].

Moreover, a new swellable microneedle adhesive inspired by endoparasite mechanisms has been developed. This adhesive is composed of a poly(styrene)-block-poly(acrylic acid) tip with a non-swellable polystyrene core. The bio-inspired design allows for strong mechanical interlocking with tissues, offering robust adhesion with minimal tissue damage. Its effectiveness in surgical adhesives and tissue grafts, along with its potential for delivering bioactive therapeutics, highlights its promise for enhancing clinical treatments [77].

Table 4 provides a comparative evaluation of swellable microneedle systems, focusing on self-application and user experience enhancements. The second column integrates mixed study findings with the authors’ critical assessment, limitations, challenges, and complementary studies for each system. From pressure-indicating sensors to antimicrobial properties, these innovations aim to improve accessibility, safety, and user confidence. However, challenges, such as variability in effectiveness and manufacturing complexities, persist across various applications.

## 7. Processes Involved in the Manufacturing of Swellable Microneedles

### 7.1. 3D Printing

**Figure 6:**
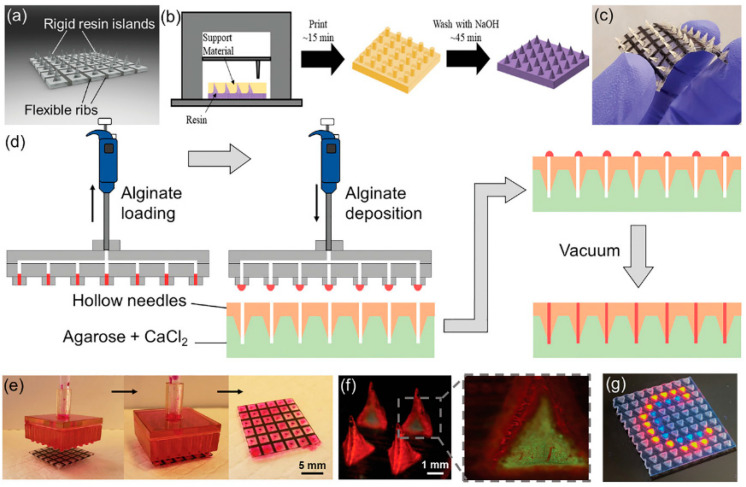
MNA fabrication and gel filling. (**a**) SolidWorks design of semiflexible MNA. (**b**) Schematic illustration of the fabrication process using 3D printing followed by cleaning with NaOH to remove support material. (**c**) Actual demonstration of semiflexible MNAs. (**d**) Concept drawing of loading mechanism for filling MNAs. (**e**) Demonstration of rhodamine-dyed alginate deposition using the loading mechanism. (**f**) Fluorescent imaging of filled needles. (**g**) Proof of concept illustrating spatial control over individual needles. Two “C”-shaped patterns in the array were filled with gels containing red and blue dyes, while the rest were filled with a transparent gel [41].

Recent advancements in 3D printing technology have significantly enhanced the fabrication of hydrogel microneedles for biomedical applications, particularly for transdermal drug delivery. This technique enables the creation of hydrogel-filled microneedle arrays that combine transdermal delivery with hydrogel encapsulation, offering precise control over microneedle geometry and allowing for the incorporation of multiple functionalities within a single device [41]. Figure 6 depicts the design and fabrication process of MNAs using a material jetting 3D printer. For instance, biodegradable polymer microneedles using fused deposition modeling (FDM) 3D printing with polylactic acid (PLA) have shown potential due to their natural degradability and the ability to achieve tip sizes as small as 1 µm through post-fabrication chemical etching. These microneedles demonstrate comparable mechanical strengths to currently fabricated microneedles and are capable of penetrating porcine skin, breaking off, and releasing small-molecule drugs over time [78].

Using reusable 3D-printed master templates with computer-aided design (CAD), hydrogel microneedles have been developed for the controlled delivery of antibiotics like amoxicillin and vancomycin. The hydrogel microneedles can be quickly and cost-effectively manufactured through micromolding [79]. The integration of digital light-processing (DLP) technology has further enhanced the 3D printing of high-precision hydrogel microneedle patches. A novel photo-printable ink comprising four functionally diverse monomers, crosslinked by aluminum hydroxide nanoparticles (AH NPs), has been formulated to create nanocomposite hydrogels with exceptional mechanical strength. This innovative approach allows for the customization of microneedle geometry and dimensions, enabling smart responsiveness to pH, temperature, and glucose levels. This adaptability facilitates more precise on-demand drug delivery, demonstrating the broad potential of hydrogel microneedles in transdermal drug delivery applications [80].

Notably, 3D-printed hydrogel microneedle arrays have also been developed for the extraction of interstitial fluid biomarkers, facilitating subsequent colorimetric detection [56]. Furthermore, 3D printing technology enables the rapid creation of complex microneedle prototypes with high accuracy, highlighting its potential for advanced transdermal drug delivery systems and customizable microneedle devices [81].

Overall, 3D printing technology offers a versatile and efficient method for producing swellable microneedles capable of enhancing transdermal drug delivery systems and providing new therapeutic strategies for conditions like alopecia and chronic wounds. The precision, customization, and multifunctionality enabled by 3D printing hold significant promise for the future of biomedical applications.

### 7.2. Molding, Micromolding, and Injection Molding

Molding, micromolding, and injection molding are versatile and effective techniques used for fabricating hydrogel-forming microneedle arrays, enabling precise control over microneedle properties and functionalities. These methods have been successfully employed to create hydrogel microneedles for various medical applications, including on-demand drug delivery, sustained drug release, and wound healing.

Micromolding: Micromolding is a versatile technique that allows for precise control over microneedle properties. One study demonstrated the fabrication of light-responsive hydrogel-forming microneedle arrays using micromolding capable of delivering ibuprofen upon exposure to light, thus enabling controlled, on-demand drug release [9]. Another study introduced a tip-swellable microneedle array patch (TSMAP) for the sustained delivery of methotrexate (MTX) in psoriasis treatment using photo-crosslinked methacrylated hyaluronic acid (MeHA) and biocompatible resin, ensuring efficient drug loading and minimal wastage [47]. Research has also explored hydrogel swelling to trigger the release of biodegradable polymer microneedles, showing sustained drug release and effective delivery of model drugs by micromolding poly-lactic-co-glycolic acid (PLGA) after filling the cavities of the mold with hydrogel microparticles [82]. Additionally, gelatin methacryloyl hydrogel microneedles (GelMA-MNs) were developed using the micromolding method for the transdermal delivery of metformin in diabetic rats, demonstrating excellent mechanical properties, moisture resistance, and improved therapeutic outcomes for diabetes [83].

Molding: Molding is commonly used for fabricating hydrogel microneedles, allowing control over disassembly times and drug release profiles. PVA-based hydrogel microneedle patches have been created with adjustable disassembly times [18], and hydrogel-forming microneedles for the transdermal delivery of acyclovir have shown sustained release over 24 h [39]. For diabetic wound healing, a glucose-responsive insulin-releasing hydrogel microneedle dressing was developed, comprising gelatin methacrylate (GelMa), glucose-responsive monomer AFPBA, and gluconic insulin. These microneedles exhibited good mechanical properties, biocompatibility, and glucose-responsive insulin release, enhancing collagen deposition and blood glucose control in animal models, making them effective for managing diabetic wounds and other chronic skin injuries [66].

Injection Molding: Injection molding is used for the scalable production of hydrogel-forming microneedle arrays, ensuring consistency and scalability. This method often involves custom metal master templates. For instance, polymeric microneedles fabricated using microinjection molding with Topas^®^ COC demonstrate feasibility for mass production at a low cost, making them suitable for biomedical applications [84]. Additionally, microneedles with through-hole capillaries have been fabricated using high-aspect-ratio microinjection molding followed by laser drilling techniques, indicating potential for advanced drug delivery systems [85].

Molding, micromolding, and injection molding enable the production of sophisticated and multifunctional microneedle arrays with precise control over drug delivery profiles, mechanical properties, and biocompatibility. These techniques are valuable for developing advanced transdermal drug delivery systems and wound healing solutions, offering tailored properties for specific medical applications.

### 7.3. Photothermal Microneedle Etching

An advanced technique, photothermal microneedle etching uses a focused 1064 nm laser beam to achieve 3D control of melting topography within a thick agarose layer. This enables the formation of spheroid clusters of specific cells from isolated single cells without physical contact [17].

### 7.4. Two-Layer Fabrication Strategy

This strategy involves creating smart microneedles using certain materials, such as silk fibroin and semi-interpenetrating network hydrogels. The two-layer structure enhances the functionality of the microneedles, particularly for glucose-responsive insulin delivery [11].

Recently, a novel phenylboronic acid (PBA)-gel-containing MN technology has gained significant research attention for its acute and sustained glucose-sensitive functionality. The PVA-coated MN patch features an interconnected porous gel drug reservoir for enhanced skin penetration efficiency and mechanical strength. This hybrid MN patch displays a “cake-like” two-layer structure, with a boronate-containing smart gel tip attached to a porous gel layer serving as a drug reservoir. The porous structure supports skin insertion and insulin loading, while the PVA coating enhances mechanical strength. Compared to MN patches with hollow drug reservoirs, this design penetrates the skin more effectively and is promising for on-demand, long-acting transdermal insulin delivery with increased patient compliance [42].

### 7.5. Drop/Dry Procedure

A simple yet effective method, the drop/dry procedure is used to fabricate PBA-based hydrogel microneedle patches for glucose-dependent insulin delivery, incorporating glucose-responsive elements within the microneedles [43]. This procedure also applies to bullet-shaped, double-layered MN arrays with water-swellable tips. These MNs interlock with soft tissues via selective distal swelling post-insertion, enabling prolonged protein release through passive diffusion. The optimal geometry of bullet-shaped MNs ensures significant adhesion strength (~1.6 N cm−2) with rat skin. Insulin-loaded MN patches using this method demonstrated a 60% insulin release over 12 h, with preserved structural integrity of the insulin. An in vivo pilot study confirmed prolonged insulin release, leading to a gradual decrease in blood glucose levels. This self-adherent transdermal MN platform is suitable for various protein drugs requiring sustained release kinetics [86].

### 7.6. Crosslinking Techniques

Various crosslinking processes enhance the mechanical properties and functionality of hydrogel microneedles.

- Physical Crosslinking: Used to produce hydrogel-forming microneedles with a poly(ethylene glycol) reservoir for albendazole delivery [36]. Citric acid crosslinking at elevated temperatures has also been employed for sustained methotrexate delivery [16].
- Chemical Crosslinking: This prevalent method creates swellable microneedles, such as hydrogel MNs based on cerium–metal organic framework composite nanozymes, for biomarker detection [24]. Additionally, hydrogel MNs integrated with aptamer probes and fluorescence detection for reagentless biomarker quantification have been developed using chemical attachment during crosslinking [59].
- Microwave-Assisted Crosslinking: A novel technique for preparing hydrogel-forming MN arrays for transdermal drug delivery, offering rapid and efficient crosslinking [34].
- UV Irradiation: Utilized for the crosslinking of methacrylated hyaluronic acid to produce swellable MN patches designed for rapid extraction of skin interstitial fluid for metabolic analysis [23]. A glucose-responsive insulin-releasing hydrogel for MN dressing fabrication developed using biocompatible gelatin methacrylate (GelMa), glucose-responsive monomer AFPBA, and gluconic insulin demonstrated adequate mechanical properties and high biocompatibility. A specific method involved preparing a PDMS-negative mold by pouring PDMS over a stainless-steel MN master structure, followed by degassing and curing. The MeHA polymer and photo-initiator solution was cast into the PDMS mold and centrifuged to fill the needle cavities. After drying, the MeHA MN patches were exposed to UV light for crosslinking, resulting in variously crosslinked MeHA MN patches with different durations of UV exposure [66].

In conclusion, the various manufacturing methods for swellable microneedles each offer unique advantages and capabilities, contributing to the development of sophisticated drug delivery systems and enhancing the potential for personalized medical applications.

## 8. Tests and Assessments

To advance the development of swellable microneedle (MN) technologies effectively, researchers must undertake a comprehensive array of tests to optimize their development and confirm their efficacy. These evaluations are crucial to ensuring that the MNs are safe, effective, and ready for clinical use.

Fabrication and Mechanical Integrity: Establishing robust methods for the fabrication of swellable MNs is imperative. Researchers should explore and assess various manufacturing techniques, including 3D printing, micromolding, and injection molding, to identify the best approach for achieving optimal mechanical properties and consistent product quality [6,9,18,41,46,47,56]. Mechanical strength and skin penetration tests are essential and must be rigorously executed to confirm that the MNs can withstand insertion forces without breaking and are capable of effectively penetrating the skin [15,16,28,35,36,39,42,44]. Employing texture analyzers to gauge force resistance and using skin-mimicking materials for insertion tests are recommended practices [8].

Drug Delivery and Release Kinetics: In drug delivery applications, understanding the release kinetics of actives from MNs is crucial. Both in vitro and in vivo studies are necessary to evaluate drug release following skin penetration [9,10,11,13,28,38,41,43,46,76]. Controlled and sustained release studies are particularly vital for chronic disease management, ensuring that drugs are delivered at therapeutic levels over extended periods without the need for frequent reapplication [16,39,41,42,47,52,64].

Biocompatibility and Safety: Biocompatibility testing is critical to confirm that MNs do not induce adverse reactions in the body. Standard development processes should include cytotoxicity assays and comprehensive biocompatibility studies [13,14,18,27,31,50,52,64]. Additionally, conducting studies with human volunteers is essential to evaluate the real-world safety and efficacy of MNs, ensuring their tolerability and effectiveness in patient use [7,22,27,62,76].

Advanced Applications and Sensing Capabilities: For more advanced applications, integrating MNs with biosensors can significantly enhance their functionality by enabling real-time detection of biomarkers, such as glucose or specific nucleic acids [19,21,24,25,29,57,59,61,63]. This integration fosters the development of responsive MN systems that adapt drug delivery based on physiological changes. Additionally, refining sampling and extraction processes is crucial for effectively utilizing MNs in diagnostic applications [20,23,58,60].

Regulatory and Clinical Validation: Before clinical adoption, MNs must clear stringent regulatory and validation hurdles. It is crucial to conduct in vivo and ex vivo validation studies to demonstrate their practical efficacy and reliability [19,26,50,54,59,75]. Comparative studies evaluating swellable MNs against other delivery systems are also beneficial to benchmark their clinical performance and utility [71,72].

## 9. Outcomes, Limitations, and Future Directions

Swellable microneedles have emerged as a transformative technology in drug delivery and biomedical diagnostics, offering controlled and targeted administration of medications to specific tissue depths. This technology significantly enhances treatment outcomes for various health conditions, including psychiatric disorders, erectile dysfunction, liver fibrosis, and spinal cord injuries [28,35,39,52,53].

The innovative manufacturing and design of these microneedles are underpinned by advanced techniques, such as 3D printing, photothermal etching, and injection molding. These methods, coupled with materials like hydrogels, chitosan, and black phosphorus, enable the scalable production of intricate, biocompatible microneedle structures that are highly effective for drug delivery and biomarker extraction [6,13,17,46,56]. The biocompatibility and safety of hydrogel-based microneedles have been extensively tested, showing no prolonged skin reactions or systemic adverse effects, making them suitable for repeated use [27,76].

Beyond traditional medical uses, swellable microneedles have found diverse applications. In agriculture, they are used for biofluid extraction and analyte detection, while in biomedical contexts they are employed in photodynamic therapy, surgical closure, and chronic wound management, including treatments with antimicrobial and antioxidant properties [31,67,69,87]. These microneedles have also significantly enhanced minimally invasive therapeutic administration, including drugs like insulin, colchicine, and anti-inflammatory agents, effectively controlling drug release and minimizing adverse effects [10,50].

Equipped with advanced sensor technologies, swellable microneedles facilitate real-time, non-invasive monitoring of biomarkers, such as glucose, pH levels, and specific nucleic acids, broadening their application beyond drug delivery [19,26,29]. Their high efficiency in targeted drug delivery and biomarker extraction enhances timely metabolic analysis of interstitial fluid, thereby improving therapeutic and diagnostic outcomes [20,23,30,33]. The design features of these microneedles, such as sensor films for insertion depth confirmation and feedback mechanisms like water-filled reservoirs, ensure painless application, reduce infection risks, and improve patient compliance, making self-application more feasible [8,22,75].

Despite these advancements, several limitations persist. There is a need for improved alignment with physiological conditions, particularly for glucose-responsive applications. Variability in clinical efficacy due to differences in skin types and conditions necessitates further research and tailored solutions [27,43,47]. Ensuring the long-term stability and consistent functionality of microneedles across various environments is challenging, particularly in maintaining stability under different storage conditions and integrating them into diverse clinical and personal health scenarios [11,14,33,37,65]. Scaling up production for commercial purposes also poses significant challenges, including ensuring consistent quality, achieving cost-effectiveness, and complying with regulatory standards [6,14,21,34,88,89]. Additionally, variability in the effectiveness of drug delivery across different skin types and environmental conditions affects the uniformity of drug delivery and biomarker extraction, necessitating continuous optimization of microneedle designs [59,90].

Future research should focus on enhancing the responsiveness of microneedle patches to physiological and environmental changes, with priorities including improved glucose-dependent swelling and insulin release, and designs tailored to individual treatment needs [14,43,44]. Expanding biomedical applications, such as delivering multiple drugs and integrating diagnostic and therapeutic functions within a single patch, could extend their use into fields like oncology or chronic disease management, where continuous monitoring and precise drug delivery are critical [9,25,49,52,53,57,63]. Innovations in materials science to enhance mechanical properties, biodegradability, and functionality are crucial for developing more robust and versatile microneedle designs [5,12,21,58,69,77].

The integration of sensor technologies for real-time monitoring and feedback within microneedle patches is expected to significantly enhance treatment efficacy and patient compliance. Further integration with digital health systems for advanced monitoring and data analytics, including real-time data transmission to healthcare providers, is anticipated to improve disease management [8,20,25,89]. Navigating regulatory pathways for quicker approvals, raising public awareness, and gaining acceptance are critical as microneedle-based treatments move toward broader clinical adoption. Addressing regulatory challenges, conducting comprehensive clinical trials to confirm efficacy and safety, and establishing clear ethical guidelines are essential for ensuring safety and public trust [27,76].

## Figures and Tables

**Table 1:** Comparative analysis of hydrogel-based MNAs for drug delivery.

Aim and Microneedle Composition	Potential Limitations and Challenges	Ref
*Scalable production of hydrogel MN arrays.* Hydrogel-forming MN arrays prepared with Gantrez^®^ S-97 and PEG 10,000 using a novel manufacturing process with custom metal MN master templates and silicone MN molds via injection molding.	Lack of discussion of clinical application; unaddressed issues in real-world use, like patient compliance and microneedle stability.	[6]
*On-demand transdermal drug delivery of ibuprofen.* MN arrays prepared from 2-hydroxyethyl methacrylate (HEMA) and ethylene glycol dimethacrylate (EGDMA) through micromolding. System loaded with up to 5% (*w*/*w*) ibuprofen included in a light-responsive 3,5-dimethoxybenzoin conjugate.	Need for external light for drug release; limitations in practical application where light exposure is inconsistent; complexity may affect manufacturing and scalability.	[9]
*Transdermal delivery of sildenafil citrate for pulmonary hypertension therapy.* Hydrogel-forming MN arrays using polyvinyl alcohol (PVA) and polyvinyl pyrrolidone (PVP) with citric acid as the crosslinking agent, integrated with sildenafil citrate (SC) tablet reservoir into a silicon microneedle mold.	Challenges in ensuring consistent drug delivery with varying patient skin types. Improvement in therapeutic efficacy and patient compliance in clinical settings needs extensive validation.	[15]
*Sustained transdermal delivery of methotrexate for rheumatoid arthritis and juvenile idiopathic arthritis.* Hydrogel-forming MN arrays from PVA/PVP crosslinked with citric acid, combined with hydroxypropyl methylcellulose (HPMC) and glycerol reservoir for methotrexate (MTX) delivery using the micromolding technique.	Long-term skin irritation potential; need for comprehensive evaluation of systemic absorption consistency.	[16]
*Transdermal drug delivery with high permeability and non-toxic properties.* PVA-based hydrogel MN patch with adjustable disassembly time prepared using a molding process. Various ratios of lower saponification (PVA6 to PVA10) were tested.	Saponification variation affects drug release and degradation times. Need for more comprehensive in vivo studies to validate safety and efficacy; variability in mechanical properties depending on saponification ratio.	[18]
*Transdermal delivery of esketamine for treatment-resistant depression.* Hydrogel-forming MN arrays in conjunction with ESK-containing reservoir. The HFMNA_s_ was prepared with Gantrez^®^ S-97, PEG 10,000 and anhydrous sodium carbonate using laser-engineered silicone micro-molds.	Potential issues with the stability and consistency of the polymeric film and lyophilized reservoirs; uncertain long-term effects and patient acceptance; challenges in achieving consistent therapeutic plasma levels.	[28]
*Efficient preparation of hydrogel MN arrays for transdermal drug delivery.* Microwave-assisted crosslinking process for hydrogel-forming MN arrays, compared with conventional thermal crosslinking. The MN prepared with aqueous solution of Gantrez^®^ S-97 and PEG 10,000.	Microwave process may have scalability limitations and could affect crosslink uniformity in larger batches; need for further in vivo validation; potential issues with patient compliance and comfort.	[34]
*Transdermal delivery of sildenafil citrate for erectile dysfunction therapy.* Hydrogel-forming microneedles (HFM) made using PVA and PVP with tartaric acid as the crosslinking agent, integrated with a polyethylene glycol (PEG) reservoir in a silicone mold and centrifugation.	Limited in vivo validation and long-term stability data for sildenafil citrate delivery challenges in ensuring consistent drug delivery and patient compliance; need for further optimization of PEG reservoir properties.	[35]
*Transdermal delivery of albendazole for cystic echinococcosis therapy.* HFM made from PVA and PVP crosslinked with citric acid, combined with a PEG reservoir for albendazole (ABZ) delivery.	Need for further optimization of PEG reservoir and crosslinking properties; variability in swelling properties affect drug delivery.	[36]
*Broadly applicable platform for transdermal drug delivery.* Methacrylated hyaluronic acid (MeHA) hydrogel MN patch with post-fabrication drug loading capability through swelling effect.	Burst release profile from the microneedles may not suit applications requiring controlled or prolonged drug release.	[37]
*Transdermal delivery of various drugs (amoxicillin, levodopa/carbidopa, levofloxacin).* Hydrogel-forming MN arrays prepared with Gantrez S-97, PEG10000, and Na_2_CO_3_, combined with directly compressed tablets (DCTs) for drug delivery in a silicone laser-engineered MN array mold.	Limited drug range due to non-aqueous reservoirs; complexity could challenge manufacturing and regulatory approval.	[38]
*Enhanced transdermal delivery of acyclovir.* Hydrogel MN arrays prepared through the micromolding method using PEG and PMVE/MA co-polymer with Na₂CO₃ for acyclovir delivery.	Potential for local skin reactions or sensitivities due to prolonged polymer exposure remains unaddressed.	[39]
*Controlled transdermal drug delivery (TDD) of ibuprofen sodium.* Super-swelling hydrogel-forming microneedles (HFMNs) made from a three-phase system (reservoir, microneedle, skin) with a mathematical model for drug transport.	Complex manufacturing may limit scalability and increase costs; accurate mathematical modeling required before production.	[40]
*Controlled transdermal delivery of various drugs.* 3D-printed microneedle arrays (MNAs) with a rigid outer layer filled with drug-eluting hydrogels (alginate, PEGDA), fabricated on a conformal backing.	Limited choice of hydrogel materials due to compatibility needs; challenges in ensuring consistent drug release profiles and mechanical strength; need for more comprehensive in vivo validation.	[41]

**Table 2:** Swellable microneedles across various treatment areas.

Aim and Microneedle Composition	Potential Limitations and Challenges	Ref
Insulin Delivery		
*Glucose-responsive insulin delivery for diabetes management.* Smart MN composed of semi-interpenetrating network hydrogel with biocompatible silk fibroin (SF) and phenylboronic acid/acrylamide. Fabricated using a two-layer strategy.	Potential for delayed or inadequate insulin release in rapid-onset hyperglycemic episodes.	[11]
*On-demand and convenient insulin delivery for diabetes.* Glucose-responsive, temperature-stable, boronate-containing hydrogel MN patch.	Effectiveness and patient adherence to self-regulated system under diverse environmental conditions need thorough investigation.	[12]
*Self-regulated insulin delivery.* Core–shell microneedle array patch with PVA, TSPBA, and CAT-NG for insulin delivery, triggered by H_2_O_2_ generated during glucose oxidation. Coated with a thin layer embedding H_2_O_2_-scavenging enzyme.	Complexity impacts manufacturing scalability and cost-effectiveness; long-term stability of enzyme activity within the gel needs thorough investigation.	[14]
*Sustained glucose-responsive transdermal insulin delivery.* Polyvinyl alcohol (PVA)-coated microneedles with an interconnected porous gel drug reservoir containing boronate for glucose-responsive insulin delivery.	Issues with boronate gel’s responsiveness to glucose fluctuations may affect insulin dosing precision under varied conditions.	[42]
*Glucose-dependent insulin delivery.* Phenylboronic-acid-based hydrogel microneedle patch prepared through copolymerization, with insulin loaded using a mild drop/dry procedure.	Performance is pH-sensitive; requires improvement to function effectively in physiological environments; limits immediate clinical application.	[43]
*Simplified preparation and glucose-responsive insulin delivery.* Crosslinking density-changeable microneedle patch made from phenylboronic-acid-grafted polyallylamine and PVA, with insulin loading by mixing with gel.	Reliability of glucose-triggered changes in crosslinking density questioned; potential inconsistency in insulin release rates with rapid glucose changes; potential issues with mechanical strength and repeated freezing/thawing process	[44]
*Insulin delivery with glucose-responsive behavior.* Hydrogel microneedle patch made from phenylboronic-acid-grafted sodium hyaluronate and PVA, with cellulose nanofiber for mechanical strength.	Long-term stability of gel’s responsiveness and durability during extended wear could be challenging.	[45]
Psoriasis Management		
*Prolonged and intelligent psoriasis management.* Detachable H(2)O(2)-responsive gel-based MN patches containing methotrexate (MTX) and epigallocatechin gallate (EGCG). EGCG used as both crosslinker and anti-inflammatory drug.	Complexity may challenge consistent manufacturing; long-term effects of continuous ROS exposure on skin health unexplored.	[10]
*Psoriasis treatment by controlling cell proliferation.* Chitosan-based MN patch fabricated using a CO_2_ laser cutter. Evaluated for delivery of Glycyrrhiza glabra extract (GgE).	Long-term effects and safety of Glycyrrhiza extract via microneedle need further investigation.	[13]
*Psoriasis treatment with photothermal-responsive capacity and real-time monitoring.* Black-phosphorus-loaded hydrogel inverse opal microneedles composed of N-isopropyl acrylamide (NIPAM)/PEGDA scaffold and gelatin/agarose filler.	Limited long-term stability and biocompatibility data for black phosphorus and photothermal materials; challenges in ensuring consistent photothermal drug release, need for more extensive clinical validation; potential issues with optical properties and real-time monitoring accuracy.	[46]
*Sustained MTX delivery for psoriasis treatment.* Tip-swellable microneedle array patch (TSMAP) made from photo-crosslinked methacrylated hyaluronic acid (MeHA) and biocompatible resin for methotrexate (MTX) delivery.	Challenges in controlling swelling and drug release rates; consistency in penetration depth across different skin types.	[47]
Pain Management		
*Treatment of chronic pain through electro-modulated analgesia*. Hydrogel-based microneedle device (EMHM) evaluated for transdermal electro-modulated analgesia.	Complexity in integrating electrical components may challenge manufacturing, cost, and user handling; could limit widespread adoption.	[48]
*Attenuation of collagen-induced arthritis.* Hydrogel microneedle (hMN) for transdermal delivery of DEK-targeting aptamer DTA6 for rheumatoid arthritis treatment.	Need for more extensive in vivo validation; potential issues with aptamer stability and efficacy in diverse patient populations; limited data on long-term joint protection.	[49]
*Management of acute gout arthritis.* Mechanically tough and ultra-swellable hydrogel microneedles (HMNs) prepared with *N*,*N*′-Bis(acrylyl)cysteamine, *N*,*N*-Methylenebisacrylamide, and Irgacure 2959, a UV-responsive crosslinker for percutaneous delivery of colchicine (Col).	Swelling capacity may cause discomfort or pressure on the skin, potentially affecting patient compliance.	[50]
Specific Therapeutic Areas		
*Inhibit fibrosis and improve cardiac function post-MI.* Gelatin methacryloyl hydrogel microneedle patch loaded with galunisertib for cardiac repair post-myocardial infarction.	Challenges in ensuring consistent drug release and cardiac repair effects; need for more comprehensive in vivo validation; potential issues with mechanical support for ventricular wall.	[51]
*Treatment of chronic liver fibrosis.* Hydrogel microneedle patch (MNP) based on GelMA for sustained release of pirfenidone (PFD) for liver fibrosis treatment.	Variability in degradation rates and drug release could affect treatment consistency; needs further validation in human studies.	[52]
*Spinal cord injury repair.* 3D-cultured MSC-derived exosome–hydrogel hybrid microneedle array patch for spinal cord repair.	Scale-up and clinical translation challenges; maintaining consistent exosome quality and performance.	[53]
*Treatment of soft tissue injuries through anti-inflammaging modulation.* Photothermal microneedle hydrogel patch with hyaluronic acid methacryloyl, LAP, and taurine-loaded Prussian blue nanoparticles for treating refractory soft tissue injuries.	Need for precise temperature control to avoid burns, or insufficient therapeutic effects could limit application outside of controlled settings.	[54]

**Table 3:** Swellable microneedles in biomedical, sensing, monitoring, and other applications.

Aim and Microneedle Composition	Potential Limitations and Challenges	Ref
*3D control of cell community topography in agarose gel.* Three-dimensional (3D) microfabrication method for agarose gel, photothermal microneedle etching (PTMNE), using a 1064 nm laser beam and photoabsorbent chromium layer.	Limited to specific types of cells and agarose gel; potential challenges in scaling up the process for larger studies or clinical applications; potential issues with cell viability and growth in the microchambers over extended periods.	[17]
*Real-time, enzyme-less electrochemical sensing of glucose.* Hydrogel MN-CGM assay with swellable dopamine–hyaluronic acid hydrogel, incorporating platinum and silver nanoparticles, and PEDOT for electrochemical sensing of glucose.	Long-term reliability and sensor performance stability, including accuracy in varying conditions, need further validation.	[19]
*Non-invasive glucose detection* via *color changes in the hydrogel film.* Wearable transdermal MN patch with photonic crystal hydrogel functionalized with phenylboronic acid for glucose monitoring.	Reliance on colorimetric analysis may limit accuracy; influenced by ambient light and user interpretation.	[20]
*Minimally invasive real-time monitoring of physiological signals.* Nanometallic conductive composite–hydrogel core–shell MN skin patch for real-time monitoring of interstitial glucose levels. Inner core coated with biomarker-specific enzymes, outer hydrogel extract biomarkers.	Integration of multiple materials and technologies may complicate manufacturing and raise costs; challenges in ensuring consistent extraction and sensing of biomarkers; could limit widespread adoption.	[21]
*Minimally invasive extraction and quantification of drugs and glucose.* MN arrays prepared from poly(methyl-vinylether-co-maleic anhydride) and poly(ethyleneglycol), crosslinked through esterification. Used for extraction and quantification of drug substances and glucose.	Potential for skin irritation or allergic reactions; variability in microneedle insertion depth and fluid uptake; need for further validation in diverse patient populations; challenges in developing mathematical algorithms for accurate blood level determination.	[22]
*Timely metabolic analysis* via *rapid ISF extraction.* Swellable microneedle patch made of methacrylated hyaluronic acid (MeHA), crosslinked through UV irradiation, for rapid ISF extraction and metabolic analysis.	Promising for rapid sampling, but the consistency and efficiency of biomarker recovery and analysis may need further evaluation.	[23]
*Rapid detection of biomarkers in ISF.* Swellable hydrogel microneedles composed of PVA and sodium alginate, integrated with cerium–metal organic frame composite nanozyme for biomarker detection.	Ensuring stability and activity of nanozyme in various conditions is challenging for consistent performance.	[24]
*Rapid lactate analysis and early melanoma diagnosis.* Bilateral core–shell microneedle patch with a shell layer for rapid ISF absorption and a core layer for lactate reaction and color change. MN prepared with PVA, lactate oxidase, and horseradish peroxidase	Dependence on visible color changes may not be reliable in all clinical conditions; challenges in interpreting results without specialized training.	[25]
*Real-time pH measurement in live animals.* Conductive hydrogel microneedle platform made from dopamine-conjugated hyaluronic acid (HA) hydrogel with PEDOT for real-time pH measurement.	Long-term stability and repeatability of pH measurements, response time, and calibration need thorough investigation for clinical applicability.	[26]
*Minimally invasive sampling and sensing of nucleic acid biomarkers.* Hydrogel-coated MN arrays with alginate–peptide nucleic acid hybrid material for sequence-specific sampling and detection of nucleic acid biomarkers from skin interstitial fluid.	Sensitivity and specificity of detecting a broad range of nucleic acid biomarkers need full validation in clinical settings.	[29]
*On-site melanoma diagnosis.* Swellable MN for extracting S100A1 from skin ISF, followed by visual quantification using antibody-conjugated magnetic microparticles and polystyrene microparticles in a microfluidic particle dam.	Complexity could pose scalability issues; accuracy of visual quantification might vary with operator experience.	[30]
*ISF biomarker extraction and colorimetric detection for chronic disease management.* 3D-printed hydrogel microneedle arrays made of crosslinked PEGDA, integrated with a multiplexed sensor for colorimetric detection of pH and glucose biomarkers.	Challenges in ensuring consistent biomarker detection and colorimetric analysis; need for more extensive in vivo validation.	[56]
*Accurate and timely sensing of levodopa for Parkinson’s disease management.* Swellable MN-mounted nanogap sensor made from hydrogel made of MeHA for single-step levodopa (LDA) sensing with redox cycling in nanogap electrodes.	Challenges in integrating nanogap sensors with microneedles include fabrication consistency, sensor durability, and system reliability in various conditions.	[57]
*Minimally invasive monitoring of blood glucose levels.* PVA/PVP hydrogel microneedle patches for ISF extraction and glucose level monitoring.	Challenges in ensuring consistent and complete recovery of ISF; maintaining accuracy in variable clinical conditions.	[58]
*On-needle measurement of biomarkers in ISF.* Hydrogel microneedles with fluorescently tagged aptamer probes for reagentless biomarker quantification.	Specificity and durability of aptamer binding in diverse clinical scenarios may affect reliability; requires further optimization.	[59]
*Minimally invasive lithium monitoring.* Hydrogel-forming microneedle arrays made from hydrolyzed poly(methyl-vinylether-co-maleic anhydride) crosslinked with PEG for lithium monitoring.	Correlation between extracted interstitial fluid lithium levels and blood serum levels needs further investigation.	[60]
*Effective ISF extraction and biomarker recovery for POCT.* Hydrogel microneedle patch made of PVA and chitosan for point-of-care testing (POCT) based on ISF.	Recovery of biomarkers from hydrogel may be challenging; might require optimization for efficient and complete extraction and analysis.	[61]
*Painless biofluid analysis and health monitoring.* Controllable swelling microneedle patch made from PEGDA/methacrylated hyaluronic acid hydrogel, combined with a paper-based sensing platform for ultrasensitive molecular recognition.	Reliance on paper-based sensors may limit detectable biomarker range and quantitative accuracy compared to standard lab equipment.	[62]
*Minimally invasive detection of clinical biomarkers.* Hydrogel microneedles combined with graphene oxide–nucleic-acid-based fluorescence biosensor for on-site detection of small molecules and proteins.	Specificity and sensitivity of the biosensor need further validation to reliably detect a wide range of biomarkers in complex biological matrices.	[63]
*Intradermal delivery for HS treatment.* Layered GelMA/PEGDA hydrogel microneedle patch loaded with compound betamethasone (CB) for hypertrophic scar treatment.	Challenges in achieving consistent corticosteroid delivery and hypertrophic scar treatment; need for more extensive in vivo validation; potential variability in drug release and efficacy.	[64]
*Promote scar-free wound healing by blocking YAP signaling.* Detachable hydrogel microneedle system with bismuth nanosheets and verteporfin for scarless wound healing.	Challenges in ensuring consistent YAP signaling inhibition and scarless wound healing; need for more comprehensive in vivo validation.	[65]
*Controlled insulin release and diabetic wound management.* Glucose-responsive hydrogel microneedle dressing made from GelMa, AFPBA, and gluconic insulin for diabetic wound healing.	Challenges in achieving consistent insulin release and diabetic wound healing; need for more extensive in vivo validation; potential variability in glucose-responsive behavior and adhesion to skin.	[66]
*Treatment of chronic wounds.* Bacterial-responsive microneedle dressing with hydrogel backing layer, composed of polycaprolactone (PCL) microspheres loaded with doxycycline hydrochloride (Dox).	Integration of multiple functionalities may complicate manufacturing and increase costs; long-term stability of the dressing needs further evaluation.	[67]
*Regenerative internal/external surgical closure.* Bio-inspired swellable hydrogel-forming double-layered adhesive microneedle patch made from mussel adhesive protein (MAP)-based shell and silk fibroin (SF)-based core.	Potential for insufficient adhesion in highly dynamic or moist internal environments; requires further optimization.	[69]
*Treatment of infectious wounds.* MgO@polydopamine nanoparticle-loaded photothermal microneedle patches combined with chitosan gel for infectious wound treatment.	Dependence on external NIR irradiation for optimal effects; potential discomfort or thermal damage from photothermal activity.	[70]
*Comparison of hydrogel-forming and dissolving microneedles for vaccine delivery.* Hydrogel-forming microneedle array for intradermal vaccination using ovalbumin as a model protein antigen.	May not deliver antigens as effectively as dissolving microneedles, which showed higher IgG titers in studies.	[71]
*Effective transcutaneous immunization.* Hydrogel patch formulation combined with microneedle arrays for transcutaneous immunization (TCI) against hepatitis B virus.	Challenges in achieving consistent antigen delivery and immune response; potential issues with antigen stability and adjuvant effects.	[72]

**Table 4:** User experience and safety of swellable microneedles.

Aim and Microneedle Composition	Potential Limitations and Challenges	Ref
*Self-application of hydrogel MN arrays.* Hydrogel-forming MN arrays tested for self-application by human volunteers with pharmacist intervention and information leaflet. Assessed skin barrier disruption and penetration depth.	Variability in self-application effectiveness across wider, less controlled populations; long-term effects of repeated self-application need thorough evaluation.	[7]
*Provide feedback on MN insertion in vivo.* Hydrogel-forming MN arrays combined with a pressure-indicating sensor film. Self-applied by human volunteers and assessed using optical coherence tomography and colorimetric analysis.	Reliance on visual color change could be subjective; may not guarantee optimal drug delivery if penetration depth is insufficient.	[8]
*Safe and repeatable clinical monitoring and treatment.* Hydrogel-forming MN array patches for clinical monitoring and skin cancer management. Evaluated repeat application in human volunteers, monitoring systemic biomarkers.	Continuous long-term studies needed to fully understand implications of chronic use, especially in varying environmental conditions.	[27]
*Improve patient acceptance and ensure consistent skin insertion.* Hydrogel-forming microneedle array patches with a water-filled reservoir for feedback mechanism.	Feedback mechanism may complicate manufacturing and increase costs; robustness of reservoir needs further validation.	[75]
*Enhanced patient safety through antimicrobial properties.* Hydrogel-forming microneedle arrays with antimicrobial properties made from hydrolyzed poly(methyl-vinylether-co-maleic anhydride) crosslinked with PEG.	Need for specific pharmacopeial standards for microneedle products; regulatory gaps can affect clinical adoption speed.	[76]
*Achieve significant adhesion to soft tissues with minimal tissue damage.* Bio-inspired swellable microneedle adhesive made from poly(styrene)-block-poly(acrylic acid) tip and polystyrene core.	Specific swelling behavior might vary under different environmental conditions; potential issues with universal applicability and bioactive therapeutic delivery	[77]

## Data Availability

Not applicable.
